# Severe Acute Hepatic Dysfunction Induced by Ammonium Acetate Treatment Results in Choroid Plexus Swelling and Ventricle Enlargement in the Brain

**DOI:** 10.3390/ijms23042010

**Published:** 2022-02-11

**Authors:** Kazuhiko Nakadate, Sumito Kamata

**Affiliations:** Department of Basic Science, Educational and Research Center for Pharmacy, Meiji Pharmaceutical University, 2-522-1 Noshio, Kiyose 204-8588, Tokyo, Japan; meijipharm.nakadatelab@gmail.com

**Keywords:** hepatic encephalopathy, choroid plexus, brain edema, aquaporin, subepithelial layer

## Abstract

Hepatic encephalopathy is a major cause of liver failure. However, the pathophysiological role of ventricle enlargement in brain edema remains unclear. Here, we used an acute hepatic encephalopathy mouse model to examine the sequential pathological changes in the brain associated with this condition. We collected tissue samples from experimental animals treated with ammonium acetate at 3 and 24 h post-injection. Despite the normalization of the animal’s ammonia levels, samples taken at 24 h after injection exhibited distinct enlargement of lateral ventricles. The choroid plexus samples obtained at 3 h post-ammonium acetate treatment indicated enlargement; however, this swelling was reduced at the later timepoint. The aquaporin-1 proteins that regulate the choroid plexus were localized both in the apical membrane and the cytoplasm of the epithelia in the control; however, they translocated to the apical membranes of the epithelia in response to ammonia treatment. Therefore, severe acute hepatic encephalopathy induced by ammonium acetate administration caused enlargement of the ventricles, through swelling of the choroid plexus and aquaporin-1 transport and aggregation within the apical membranes.

## 1. Introduction

Hepatic encephalopathy (HE) is a major cause of liver failure, frequently associated with the progression of end-stage liver disease [1]. In addition, liver failure is closely associated with the development of intracranial hypertension, which commonly manifests as brain edema; this, in turn, plays a critical role in the development of encephalopathy. HE exists as a spectrum from minimal dysfunction to coma and may arise from ammonia-associated neurotoxicity, whereby metabolic dysfunction leads to glutamine accumulation, astrocyte swelling and nitric oxide-induced vasodilation [2]. Elevated intracranial pressure, secondary to cerebral edema, is common in liver failure and occurs in 80% of comatose patients [2,3,4]. In patients with acute HE, brain edema plays a crucial role in the associated neurological deterioration. Fluid entry from the vascular compartment into the brain increases the brain volume, in turn increasing the intracranial pressure [1]. Vasogenic edema occurs mainly due to the breakdown of the tight endothelial junctions that make up the blood–brain barrier (BBB) [5]. The disruption in the cellular metabolism impairs functioning of the sodium and potassium pumps in the glial cell membrane surrounding the blood vessels. This causes the accumulation of osmotically active molecules, leading to cellular retention of sodium and water and consequently glial cell swelling and neuronal cytotoxic edema [5,6,7]. The molecular mechanisms leading to glial cell swelling are not fully understood. They are believed to be linked to the osmo-sensitive or stretch-sensitive intracellular signaling cascades, involving [Ca^2+^]i transients, aquaporins (AQPs; mainly AQP4) and volume-regulated anion channels [7,8,9,10].

Cerebral magnetic resonance imaging (MRI) or computer tomography (CT) is used to detect changes in magnetization transfer imaging ratios in patients with HE, and these are interpreted as indicators of an increase in brain water content [11,12,13]. However, until the 1990s, both MRI and CT measurements of both the brain and ventricular volumes were not sensitive enough for direct, serial measurements of small changes in brain size in patients with HE, who have structurally normal brains. However, subsequent improvements in both MRI and CT techniques have facilitated more nuanced evaluations of very small differences in both brain and ventricular volumes, which may not be obvious during standard radiological inspection [14]. Therefore, current guidelines promote the use of both MRI- and CT-based imaging of patients with HE. These evaluations have revealed that ventricular enlargement is also common in patients with minimal hepatic encephalopathy [15] and that there are acute changes in ventricular volume during the treatment of HE patients [16]. These studies also reported elevated intracranial pressure, cerebral edema and ventricular volume changes in HE patients with increased blood ammonia levels. 

Although experimental data suggests that elevated intracranial pressure is likely to induce hydrocephaly [17,18], there have been no clinical reports describing severe hydrocephaly in response to elevated blood ammonia levels. Furthermore, the mechanism by which elevated blood ammonia levels may induce hydrocephaly remain unclear. Therefore, we designed this study to use both histopathological and ultra-microstructural evaluations to determine whether a rapid and acute increase in blood ammonia levels may induce hydrocephalus in a murine model of severe HE. We also evaluated changes in the expression and distribution of aquaporins in the choroidal plexus of these animals revealing the likely mechanism by which elevated blood ammonia levels increase cerebrospinal fluid production and encourage hydrocephaly.

## 2. Results

### 2.1. Biochemical Analyses

**Figure 1:**
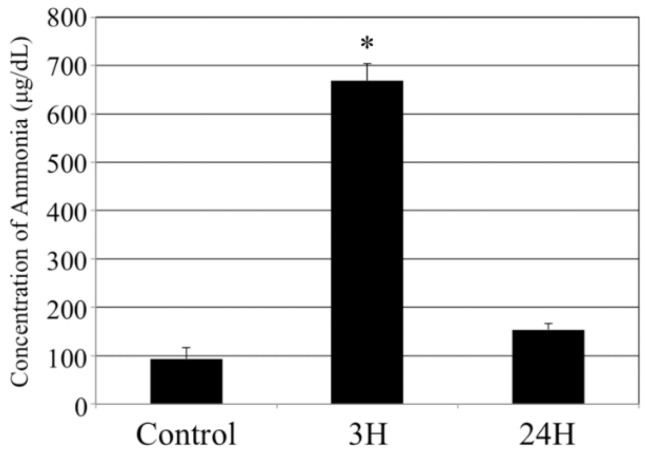
Control, 3H and 24H indicate the average blood ammonia concentration of the animals in the vehicle treatment control group and the treated animals at 3 and 24 h after ammonia treatment, respectively. Data are expressed as mean (each animal number = 4) ± standard error. *: *p* < 0.05 vs. control.

In this study, we investigated whether the brain is affected by acute hepatic dysfunction. First, we evaluated the sequential changes in the blood ammonia concentration in each animal following treatment with the ammonia inducer compound (Figure 1). The average blood ammonia in the vehicle-administered control animals was low (93.7 ± 22.7 μg/dL); however, it increased in the treated animals at 3 h post-injection (669.0 ± 34.8 μg/dL, *p* < 0.05), before returning to almost control levels at 24 h post-injection (154.0 ± 12.2 μg/dL). 

We evaluated various biochemical parameters in these groups using commercial kits. The total protein content was significantly decreased at 3 h post-ammonia administration when compared to that in the control (3 h post-ammonia treatment; 2.79 ± 0.32 g/dL vs. control; 3.63 ± 0.25 g/dL). In addition, the evaluation of the animals at 24 h post-ammonia treatment revealed that while the total protein had returned to control levels (3.83 ± 0.35 g/dL), the albumin levels (control; 1.57 ± 0.25 g/dL, 3 h post-ammonia treatment; 1.70 ± 0.33 g/dL, 24 h post-ammonia treatment; 1.87 ± 0.25 g/dL) remained unchanged.

### 2.2. Sequential Macroscopic Anatomical Changes in the Brain following Hepatic Encephalopathy

**Figure 2:**
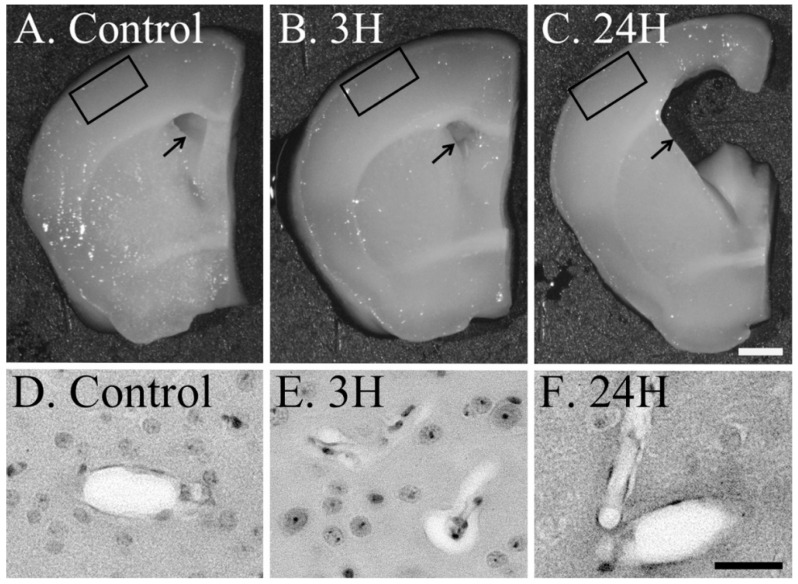
Control (**A**), 3H (**B**) and 24H (**C**) representative images of the brain tissues from the vehicle control animals (at 3 h) and those from animals sacrificed at 3 h and 24 h after ammonia treatment, respectively. Control (**D**), 3H (**E**) and 24H (**F**) illustrate the perivascular edema of the cerebral cortex layer II/III (each black square in A through C). Arrows indicate the position of the lateral ventricle and the scale bars in C and F = 1 mm and 200 μm, respectively.

We evaluated the anatomical changes in the brain in response to acute hepatic dysfunction (Figure 2). The brains of the control (both 3 h and 24 h post-vehicle treatment groups) and 3 h post-treatment groups appeared healthy (Figure 2A,B); however, the brains from the 24 h post-treatment group demonstrated significant alterations in response to acute hepatic dysfunction (Figure 2C). All the lateral ventricles were enlarged (arrows in Figure 2A–C), as were the third and fourth ventricles (data not shown). Therefore, acute hepatic dysfunction caused by ammonia toxicity induced the enlargement of the ventricle. The perivascular swelling of the cerebral cortex layer II/III were observed in the 3 h post-ammonia treatment groups (Figure 2E) as previous studies, but not in the control and 24 h post-ammonia treatment groups (Figure 2D,F).

### 2.3. Histopathological Evaluation of the Changes in the Choroid Plexus in Response to Hepatic Encephalopathy

**Figure 3:**
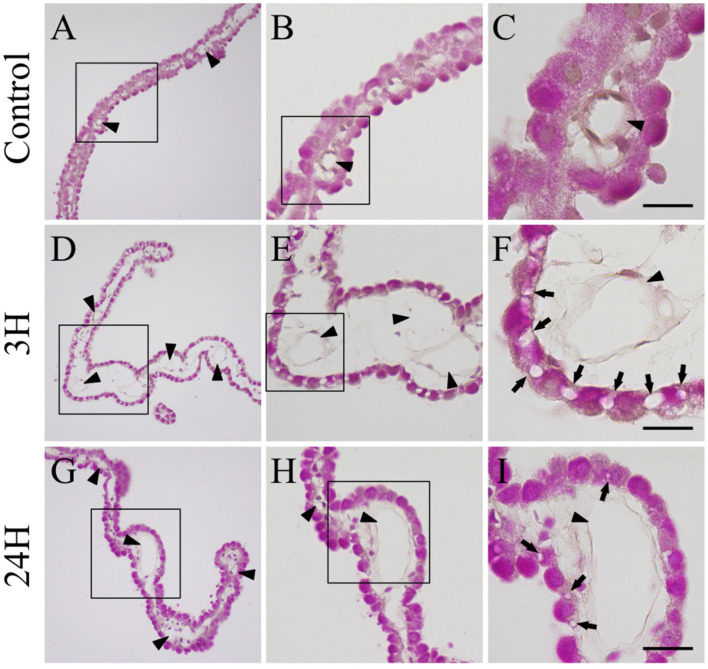
Sequential histopathological changes in the choroid plexus after hepatic encephalopathy (*n* = 3). Control (**A**–**C**), 3H (**D**–**F**) and 24H (**G**–I) indicate representative images for the brain tissues from vehicle-treated animals (at 3 h) and from animals at 3 h and 24 h after ammonia treatment, respectively. **A**,**D**,**G** are low-magnification views of the hematoxylin-eosin stained choroid plexus. **B**,**E**,**H** are enlarged images of the sections highlighted in black boxes in **A**,**D**,**G**, respectively. **C**,**F**,**I** show enlarged views of the black boxes in **B**,**E**,**H**, respectively, and arrowheads indicate blood vessels while arrows indicate separated epithelial cells. Scale bars = 10 μm.

We evaluated the histopathology of the choroid plexus (Figure 3). The choroid plexus from both the control animals (3 h and 24 h after vehicle treatment) were normal and thin (Figure 3A–C), with normal epithelia and vascular structures (arrowheads in Figure 3A–C). These tissues also presented with a uniform eosin stain, highlighting the clearly interlocked structure of their epithelium. There were no significant differences between the vehicle administered control animals at 3 h and 24 h post-administration. 

The choroid plexus from the 3 h post-treatment group demonstrated clear swelling when compared to that from the control (Figure 3D–F), with both the capillaries (arrowheads in Figure 3D–F) and loose connective tissues indicating some degree of expansion. Increased magnification (Figure 3F) revealed a more erratic eosin staining profile, indicating that the epithelia had begun to thin and expand, creating gaps in the epithelium (arrows in Figure 3F). This swelling was still prevalent in the samples from the 24 h post-treatment group (Figure 3G–I), although it was significantly reduced when compared to that in the 3 h samples. In addition, the capillary and loose connective tissue networks had returned to normal state and the eosin staining was uniformly distributed. Many of the epithelial cells had returned to normal, with most gaps reduced back to tight junctions. However, some intercellular gaps remained (arrows in Figure 3I).

### 2.4. Ultrastructural Changes in the Choroid Plexus in Response to Hepatic Encephalopathy

**Figure 4:**
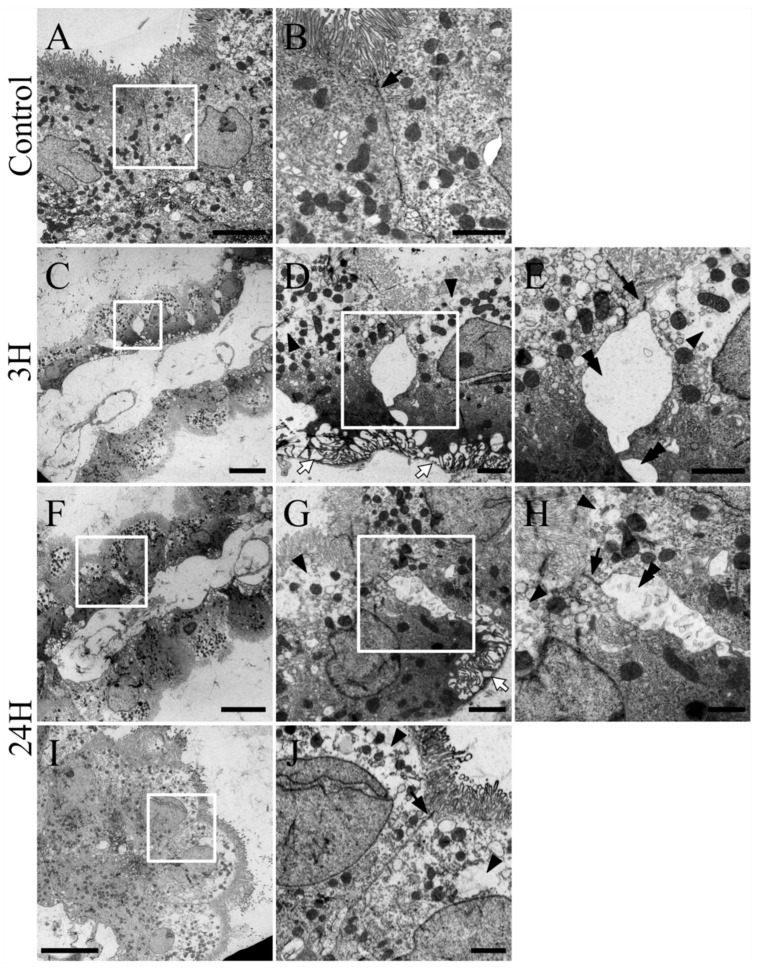
Control (**A**,**B**), 3H (**C**–**E**) and 24H (**F**–**J**) are representative images of the tissues collected from vehicle treated animals (*n* = 6) and those collected from animals at 3 h (*n* = 3) and 24 h (*n* = 3) post-ammonia treatment, respectively. Pictures **B**,**D**,**E**,**G**,**H**,**J** are the enlarged image of the tissue sections highlighted in white boxes in **A**,**C**,**D**,**F**,**G**,**I**, respectively. **E**,**H** are the highest magnification views of the white boxes from **D**,**G**, respectively. Arrowheads indicate blood vessels, while black arrows indicate the tight junctions between the epithelial cells. White arrows show the near basal membrane swelling in these epithelial cells and the arrowheads indicate epithelial cell swelling. Double arrowheads indicate separated epithelial cells. Scale bars in **C**,**F**,**I** = 10 μm, in **A**,**D**,**G** = 5 μm and in **B**,**E**,**H**,**J** = 2 μm.

We evaluated the changes in the choroid plexus using electron microscopy (Figure 4). The choroid plexus of both control animals (3 h and 24 h after vehicle treatment) were healthy (Figure 4A), with uniform distribution of their intracellular structures and normal cell–cell junctions (arrows in Figure 4B). However, as in the histopathology evaluations, the choroid plexus of animals from the 3 h post-ammonia treatment group (*n* = 3) exhibited distinct swelling (Figure 4C–E) with expansion in the capillaries and loose connective tissues (Figure 4C). The epithelia of the choroid plexus were expanded and thinning, and the intracellular density of the epithelia was less uniform, with an increase in high-density components on the basal membrane side and low-density or watery substances on the apical side (arrowheads in Figure 4D,E). These results were similar to those of the HE staining. In addition, there was an elongation in many of the structures located near the basal membrane side of the choroid plexus (white arrows in Figure 4D). Increased magnification revealed many gaps in the epithelia of these samples (double arrowheads in Figure 4E). The junctional structures remained relatively normal (arrow in Figure 3F). 

Ultrastructural evaluation revealed swelling in the choroid plexus of animals in the 24 h-post-ammonia treatment group (*n* = 3, Figure 4F–J), with many of the abnormalities (Figure 4F–H) being similar to those observed in the 3 h post-treatment samples. However, parts of the choroid plexus (Figure 4I,J) exhibited some degree of improvement compared to that in the 3 h tissues. There was a reversal in the intracellular density of the epithelia, although some epithelial cells still presented with low-density regions (arrowheads in Figure 4J). Many epithelia were normal and most of the gaps in these layers had been closed, while the junctional structures remained intact (arrow in Figure 4J).

### 2.5. Changes in the Distribution of Aquaporin 1 across the Choroid Plexus after Hepatic Encephalopathy

**Figure 5:**
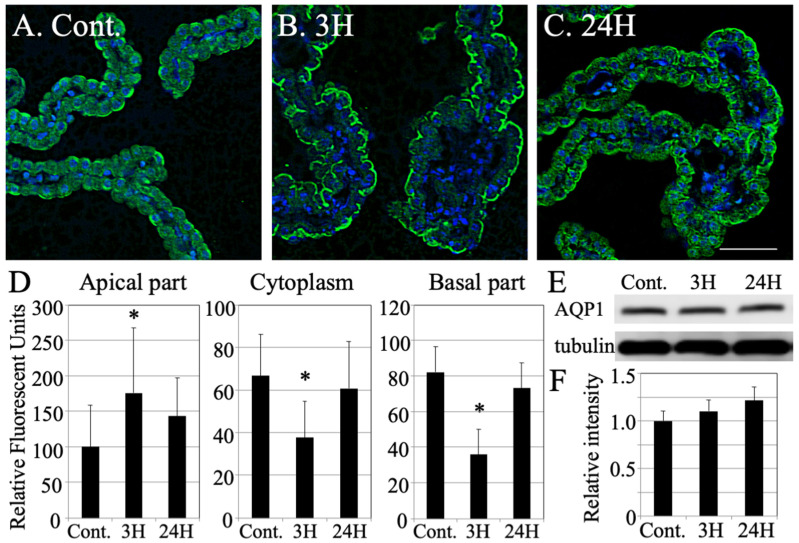
Changes in the distribution and expression of AQP1 in choroid plexus tissues following hepatic encephalopathy. (**A**–**C**) Immunohistochemical staining for aquaporin-1 (green color) and DAPI stained nucleus (blue color) in each animal (each *n* = 3). (**D**) Relative intensity of aquaporin-1 (green color) in the apical portion of the choroid plexus, cytoplasm of the choroid plexus and basal part of the choroid plexus as determined using densitometry. Data are expressed as mean ± standard error. All data are shown as the relative intensity (100 = the intensity of the apical section in the control). (**E**) Western blot revealing the timing of aquaporin-1 expression. (**F**) Densitometry analysis of the Western blot data (each number = 3). Data are expressed as mean ± standard error. Data are shown as the relative intensity (100 = the intensity of control value). Cont., 3H and 24H represent the various treatment groups, as previously described. *: *p* < 0.05 compared with the control.

We speculated that there was an increase in the outflow of cerebrospinal fluid (CSF) from these tissues. This was subsequently confirmed through evaluations of the aquaporin 1 (AQP1) expression pattern (Figure 5). In the control choroid plexus samples, AQP1 was expressed in both the apical and basal membranes of the epithelia and within the cytoplasm (Figure 5A). However, in the tissues from the 3 h -ammonia treatment animals (*n* = 3), AQP1 accumulated in the apical membranes of the epithelia, while disappearing from the cytoplasm and basal membranes (Figure 5B). This expression pattern reverted to the original expression pattern in samples from the 24 h post-ammonia treatment group (*n* = 3, Figure 5C). Next, we measured the change in AQP1 intensity following hepatic encephalopathy using densitometry. This revealed a transient increase in AQP1 in the apical membranes in response to ammonia treatment (Figure 5D) and a corresponding decrease in its expression in the cytoplasm and basal membranes. 

Finally, we evaluated the protein response of AQP1 during hepatic encephalopathy using Western blot analysis (each number = 3, Figure 5E). The optical densities of the bands showed a slight increase over time but did not change significantly (Figure 5F). These results indicate that there was no induction of AQP1 in response to ammonia treatment suggesting that AQP1 proteins were transported and/or aggregated in the apical part of the cell membrane and sequestered from the cytoplasm and basal cell membrane.

## 3. Discussion

Ammonia is usually produced in the gastrointestinal tract via the degradation of proteins and amino acids by both host and bacterial enzymes. This ammonia then enters the portal circulation and is metabolized in the liver to urea and glutamine before excretion. When the liver is dysfunctional, the blood ammonia concentration increases. This allows ammonia to enter the astrocytes, increasing their volume and inducing brain edema [19]. The normal blood ammonia concentration is 30–80 μg/dL (9–35 μmol/L) and the reference range for blood ammonia concentration decreases with age [20,21]. The risk of cerebral edema increases when the arterial ammonia levels exceed 340 μg/dL (200 μmol/L) [20,21]. HE patients present with increased blood ammonia concentrations; this often manifests in their clinical presentation, including that in a high degree of comatose patients and those presenting with brain edema [21]. We designed this study to investigate how increased blood ammonia concentrations affect the brain, using a newly established murine model. In this study, animals were treated with two doses of ammonia before falling into a coma for several hours and then awakening within 6 h of the 2nd dose and returning to normal function within 1 h of waking. This conditional change in the mice correlates with a transient increase in blood ammonia levels. In addition, these treatments induced a transient decrease in total protein levels, which reverted to normal levels after 24 h. This suggested that the liver function in the animals had partially recovered or that the systemic management was becoming normal. The albumin levels remained unchanged; therefore, it is possible that these animals maintained oncotic pressure.

Macro-analysis of the brain and HE-staining revealed cerebral edema in these animals; this included swelling of the tissues around the blood vessels in the cerebral cortex of the brain. This result is consistent with that from previous studies [21]. The subsequent HE-stained images of the cerebral cortex, taken 24 h after the administration of ammonia, showed that the swelling around the blood vessels was significantly reduced, suggesting a concurrent reduction in the edema of the parenchyma. However, macro-analysis revealed enlargement of the ventricles; it was the most pronounced in the lateral ventricle. In addition, histological and ultrastructural analysis of the choroid plexus revealed significant swelling of the interstitium between the choroid plexus epithelial cells at 3 h post-ammonia administration, suggesting an increase in the interstitial fluid (ISF) volume in these animals. The space at the interepithelial site was also expanded. We evaluated these results considering the function of the choroid plexus [22,23,24,25]. Despite the continued functioning of the blood-cerebrospinal fluid barrier (BCSFB) in this model animal, this swelling could result in a delay in the passage of many substances, including water molecules, across the epithelium. This swelling was reduced 24 h post-ammonia administration, indicating that the substance transfer to cerebrospinal fluid (CSF) was enhanced. When we combine these results with those of a previous study [26], ventricular enlargement could be the result of enhanced material transfer from the choroid plexus to the CFS.

Previous studies of hepatic encephalopathy have reported swelling around the blood vessels that make up the BBB in the cerebral cortex of the brain; aquaporin 4 is closely linked to the activity of astrocytes around these blood vessels [27,28,29,30,31,32]. We detected a similar swelling around the blood vessels in the cerebral cortex of the brain following the administration of ammonium acetate. Therefore, we analyzed the changes in the expression pattern of aquaporin 1 using immunohistochemistry. The expression of aquaporin 1 in the apical membrane of the choroid plexus increased in response to increase in blood ammonia levels. However, there was no significant difference in the total expression level of AQP1, suggesting that aquaporin 1 was transported from the cytoplasm to the apical membrane in response to ammonia toxicity. Such dynamic changes in the distribution pattern of aquaporin 1 suggest that the choroid plexus mediates this transient mass transfer. Aquaporin 1 plays an important role in the onset of hydrocephalus [33,34]. Future studies would clarify the more remarkable changes in the brain associated with HE, through evaluating aquaporin 1, which, as a central component of the water channels, is likely to be involved in the development of hydrocephalus [35,36] and the various transporters involved in the transfer of other substances [37]. 

The increase in the blood ammonia levels in response to advancing liver disease, including hepatic encephalopathy, results in almost all patients becoming comatose; this specific factor is closely monitored, and preventative therapies are introduced early. Therefore, it is rare for any human patient to reach the high levels of ammonia described in this study; however, even at lower ammonia concentrations, most patients present with some ventricular enlargement [15,16]. There is an increasing number of patients with various liver dysfunctions, including HE; therefore, it is becoming increasingly important to understand and evaluate the pathological effects of these conditions on the brain. This study will aid in the early diagnosis of various brain diseases associated with liver dysfunction, including HE, which is likely to increase in the future. It would facilitate the introduction and development of early treatment methods.

## 4. Materials and Methods

### 4.1. Animals

We used 42 C57BL/6J male mice (10 weeks old; Charles River, Japan). The animal experiments were performed in accordance with the National Institutes of Health Guide for the Care and Use of Laboratory Animals and our protocol was approved by the Laboratory Animal Ethics Committee of Meiji Pharmaceutical University (No. 2704, 1 April 2017). All efforts were made to minimize animal suffering and reduce the number of animals used in the study. 

### 4.2. Inducing Acute Liver Failure Induction 

Twenty four 10-week-old C57BL/6J mice were treated intraperitoneally with ammonium acetate (4.5 mmol/kg body weight, Sigma-Aldrich, MO, USA) using two intraperitoneal injections with a 15 min interval between them. Eighteen age-matched male mice were injected intraperitoneally with saline and used as the control.

### 4.3. Biochemical Analysis

Blood samples were taken from the tail vein of animals (4 mice per group) from all evaluation groups (3 h and 24 h after ammonium acetate or vehicle injection) and then 100 μL of each sample was diluted in an equivalent volume of heparin supplemented physiological saline. These samples were then centrifuged, and the plasma obtained after centrifugation was quickly cryopreserved at −80 °C for later evaluation. Plasma ammonia concentrations were determined using a Cica-liquid NH_3_ kit (Kanto Chemical Co., Inc. Tokyo, Japan). Total plasma protein (TP) and albumin (ALB) concentrations were determined using the appropriate test kits (Wako Pure Chemical Industries, Ltd. Osaka, Japan).

### 4.4. Western Blot Analysis

This study was designed to examine the choroid plexus in the lateral ventricle of mice following acute liver failure and all proteins were prepared for Western blotting, as described previously [38,39]. Briefly, the animals were deeply anesthetized using an overdose of sodium pentobarbital (50 mg/kg, intraperitoneally; Nembutal^®^; Abbott Laboratories, IL, USA) and then each of the four mice in each group (control, 3 h and 24 h after ammonium acetate injection) were perfused via their left ventricle with ice-cold saline; their choroid plexuses were rapidly removed before being homogenized in ice-cold homogenate buffer (20 mM Tris-HCl at pH 7.5, 1 mM ethylenediaminetetraacetic acid, 1 mM dithiothreitol and 150 mM NaCl) supplemented with protease inhibitors (1 tablet/10 mL homogenate buffer, Complete^TM^ Mini, Roche Diagnostics, Basel, Switzerland). Homogenates were centrifuged at 500× *g* for 5 min at 4 °C and the supernatants were placed in sodium dodecyl sulfate (SDS) sample buffer (62.5 mM Tris-HCl, pH 6.8, containing 3% SDS, 5% glycerol and 2% 2-mercaptoethanol) and boiled for 5 min. Protein concentrations were evaluated using the Bradford method, with a commercial protein assay kit (Bio-Rad Laboratories, Inc., Hercules, CA, USA) and quantified against bovine serum albumin standards.

Equal concentrations of protein from each group were then subject to SDS-polyacrylamide gel electrophoresis and transferred onto a polyvinylidene difluoride membrane (Immobilon^TM^-P, EMD Millipore, Billerica, MA, USA) for evaluation using an ECL-Plus immunoblotting detection system (GE Healthcare Life Sciences, Buckinghamshire, UK). These membranes were blocked using 10% (*w/v*) skim milk (Becton, Dickinson and Company, Franklin Lakes, NJ, USA) in phosphate-buffered saline (PBS) containing 0.1% Tween 20 (TPBS, pH 7.5) for 1 h at room temperature (RT). They were washed and incubated with a mouse anti-aquaporin-1 antibody (1:200 dilution in TPBS, Abcam, Cambridge, UK) for 2 h at RT. The membranes were washed again and incubated with a horseradish peroxidase-conjugated anti-mouse antibody (1:2000 dilution in TPBS, LI-COR Corporate, Lincoln, NE, USA) for 45 min at RT. Immunoreactive bands were detected using western PREMIUM chemiluminescent substrate (LI-COR Corporate, Lincoln, NE, USA) and a LI-COR C-DiGit chemiluminescence Western blot scanner (LI-COR Corporate, Lincoln, NE, USA). These blots were then stripped using the Restore^TM^ Stripping Buffer (Thermo Fisher Scientific K. K., Tokyo, Japan) and incubated with a primary antibody against α-tubulin (Sigma Aldrich, St. Louis, MO, USA), which was used as an internal loading control.

### 4.5. Tissue Preparation

Tissue preparations for both immunohistochemistry (IHC) and electron microscopy were performed, as previously described [38]. Tissue sections were collected from animals, as described earlier and they were perfused with a fixative (1450 mOsm/L) containing 4% paraformaldehyde in 0.1 M phosphate buffer (PB, pH 7.4). The animal brains were removed and post-fixed in the same fixative overnight at 4 °C and sliced into 2 mm sections using a brain slicer. The sections containing the choroid plexus of the lateral ventricle were immersed in graded concentrations of ethanol, cleared in Lemosol A (Wako Pure Chemical Industries, Ltd., Tokyo, Japan) and embedded in paraffin.

For transmission electron microscopy, the animals from each group were perfused with a fixative (1570 mOsm/L) containing 4% paraformaldehyde and 1% glutaraldehyde in 0.1 M PB (pH 7.4). The brains were quickly removed and post-fixed in the same fixative for 24 h at 4 °C before sectioning at 1 mm thickness using a brain slicer. The sections including the choroid plexus of the lateral ventricle were immersed in OsO_4_ (TAAB Laboratories, Ltd., Aldermaston, UK) for 2 h, dehydrated in ethanol and embedded in Epon-812 resin (TAAB Laboratories, Ltd., Aldermaston, UK). 

### 4.6. Histological and Immunohistochemical Analysis

Both hematoxylin-eosin (HE) staining and immunohistochemical staining required the ultratome sectioning of the paraffin-embedded brain blocks; they were cut into 5-μm-thick sections using a sliding microtome (REM-710, Yamato Kohki Industrial, Tokyo, Japan). All sections were mounted on glass slides, deparaffinized with Lemosol A and immersed in graded concentrations of ethanol and distilled water. 

Sections were stained with HE, dehydrated in graded concentrations of ethanol and cleared with Lemosol A; they were then placed on cover slips. 

For the IHC analyses, the sections were incubated in 10 mM sodium citrate buffer (pH 6.0) for 60 min at 95 °C, cooled to room temperature, washed with PBS containing Triton-X 100 and incubated with the blocking solution from the Vector M.O.M. Fluorescent Kit (Vector Laboratories, Inc., Burlingame, CA, USA). Sections were incubated with mouse anti-Aquaporin-1 antibody (1:300 dilution in PBS, Abcam, Cambridge, UK) for 2 h at RT, washed several times with PBS and incubated with biotinylated anti-mouse antibody and fluorescein avidin complex solution. The sections were washed and incubated with Hoechst 33,342 solution for counterstaining and then placed on cover slip.

HE- and immune-stained section images were captured using a CCD camera (BZ-X700, Keyence, Japan).

### 4.7. Electron Microscopy

The choroid plexus of the lateral ventricle was embedded in Epon-812 resin and trimmed under light microscopy. Ultrathin sections (70 nm thick) were cut using an MT-XL ultramicrotome (Research and Manufacturing Company, Tucson, AZ, USA) and placed onto grids (Veco, Eerbeek, The Netherlands). The electron-stained ultrathin sections were examined using transmission electron microscopy (JEM-1011, JEOL, Ltd., Tokyo, Japan) and images were captured using a CCD camera.

### 4.8. Data Analysis

The Western blot images were analyzed using Image J software (NIH, Bethesda, MD, USA). The densities of the immunoreactivity bands were quantified, and statistical analysis was performed using StatView statistical software (SAS Institute Inc., Cary, NC, USA). Differences were analyzed using analysis of variance (ANOVA) and significance was set at *p* < 0.05.

## Data Availability

Not applicable.
